# CME ACTIVITY: Acute Conjunctivitis with Episcleritis and Anterior Uveitis Linked to Adiaspiromycosis and Freshwater Sponges, Amazon Region, Brazil, 2005

**DOI:** 10.3201/eid1504.0812811

**Published:** 2009-04

Medscape, LLC is pleased to provide online continuing medical education (CME) for this journal article, allowing clinicians the opportunity to earn CME credit. This activity has been planned and implemented in accordance with the Essential Areas and policies of the Accreditation Council for Continuing Medical Education through the joint sponsorship of Medscape, LLC and Emerging Infectious Diseases. Medscape, LLC is accredited by the Accreditation Council for Continuing Medical Education (ACCME) to provide continuing medical education for physicians. Medscape, LLC designates this educational activity for a maximum of 0.5 *AMA PRA Category 1 Credits*™. Physicians should only claim credit commensurate with the extent of their participation in the activity. All other clinicians completing this activity will be issued a certificate of participation. To participate in this journal CME activity: (1) review the learning objectives and author disclosures; (2) study the education content; (3) take the post-test and/or complete the evaluation at **http://www.medscape.com/cme/eid**; (4) view/print certificate.

## Learning Objectives

Upon completion of this activity, participants will be able to:

- Describe the mechanism of infection for adiaspiromycosis.
- Identify the age group most susceptible to ocular adiaspiromycosis.
- Describe presenting symptoms associated with ocular adiaspiromycosis.
- Describe the frequency of ocular lesions associated with adiaspiromycosis.
- Identify risk factors for ocular adiaspiromycosis.

## Editor

**Beverly Merritt**, Technical Writer-Editor, *Emerging Infectious Diseases*. Disclosure: *Beverly Merritt has disclosed no relevant financial relationships*.

## CME AUTHOR

**Désirée Lie, MD, MSEd**, Clinical Professor, Family Medicine, University of California, Orange; Director, Division of Faculty Development, UCI Medical Center, Orange, California. *Disclosure: Désirée Lie, MD, MSEd, has disclosed no relevant financial relationships.*

## AUTHORS

Disclosures: **Tatiana M. Lanzieri, MD, MSc**, has disclosed that she has been employed by GlaxoSmithKline since April 2008, but this study was conducted while she was working in the Brazilian Ministry of Health. **Márcia O. Mendes, BSc, MSc; Mario A.P. Moraes, MD; Ernesto I.M. Renoiner, ND; Marta H.P. Dantas, ND, MSc; Carlos F. Fonseca, MD; Expedito J.A. Luna, MD;** and **Douglas L. Hatch, MD, MPH**, have disclosed no relevant financial relationships.

## Earning CME Credit

To obtain credit, you should first read the journal article. After reading the article, you should be able to answer the following, related, multiple-choice questions. To complete the questions and earn continuing medical education (CME) credit, please go to **http://www.medscape.com/cme/eid**. Credit cannot be obtained for tests completed on paper, although you may use the worksheet below to keep a record of your answers. You must be a registered user on Medscape.com. If you are not registered on Medscape.com, please click on the New Users: Free Registration link on the left hand side of the website to register. Only one answer is correct for each question. Once you successfully answer all post-test questions you will be able to view and/or print your certificate. For questions regarding the content of this activity, contact the accredited provider, CME@medscape.net. For technical assistance, contact CME@webmd.net. American Medical Association’s Physician’s Recognition Award (AMA PRA) credits are accepted in the US as evidence of participation in CME activities. For further information on this award, please refer to http://www.ama-assn.org/ama/pub/category/2922.html. The AMA has determined that physicians not licensed in the US who participate in this CME activity are eligible for *AMA PRA Category 1 Credits*™. Through agreements that the AMA has made with agencies in some countries, AMA PRA credit is acceptable as evidence of participation in CME activities. If you are not licensed in the US and want to obtain an AMA PRA CME credit, please complete the questions online, print the certificate and present it to your national medical association.

### Article Title: Acute Conjunctivitis with Episcleritis and Anterior Uveitis Linked to Adiaspiromycosis and Freshwater Sponges, Amazon Region, Brazil, 2005

## CME Questions

1. Which of the following is the *most* likely mechanism for disease associated with adiaspiromycosis?
  A. Conidia invasion
  B. Immune response
  C. Allergy
  D. Fungemia
2. Which of the following is the *most* common age group reported to be infected with ocular adiaspiromycosis in the initial case series of 17 patients in this article?
  A. Less than 5 years
  B. 5 to 15 years
  C. 16 to 25 years
  D. 26 to 35 years
3. Which of the following is *least* likely to be reported as an ocular-related symptom in patients with ocular disease associated with adiaspiromycosis?
  A. Conjunctival hyperemia
  B. Photophobia
  C. Blurred vision
  D. Excessive tearing
4. Which of the following *best* describes the frequency of bilateral corneal opacities in patients with confirmed ocular disease in this case series?
  A. 13%
  B. 20%
  C. 35%
  D. 80%
5. Which of the following is *least* likely to be a risk factor associated with ocular adiaspiromycosis in this case series?
  A. Diving in the Araguaia River
  B. Fishing in the Araguaia River
  C. Male gender
  D. Drinking Araguaia River water

### Activity Evaluation

**Table:** 

**1. The activity supported the learning objectives.**				
Strongly Disagree				Strongly Agree
1	2	3	4	5
**2. The material was organized clearly for learning to occur.**				
Strongly Disagree				Strongly Agree
1	2	3	4	5
**3. The content learned from this activity will impact my practice.**				
Strongly Disagree				Strongly Agree
1	2	3	4	5
**4. The activity was presented objectively and free of commercial bias.**				
Strongly Disagree				Strongly Agree
1	2	3	4	5

